# Molecular Alterations in Spermatozoa of a Family Case Living in the Land of Fires—A First Look at Possible Transgenerational Effects of Pollutants

**DOI:** 10.3390/ijms21186710

**Published:** 2020-09-13

**Authors:** Gennaro Lettieri, Federica Marra, Claudia Moriello, Marina Prisco, Tiziana Notari, Marco Trifuoggi, Antonella Giarra, Liana Bosco, Luigi Montano, Marina Piscopo

**Affiliations:** 1 Department of Biology, University of Naples Federico II, 80126 Napoli, Italy; gennarole@outlook.com (G.L.); federicamarra14@gmail.com (F.M.); cla_mar97@hotmail.it (C.M.); marina.prisco@unina.it (M.P.); 2 Check Up—Day Surgery, Polydiagnostic and Research Centre, Reproductive Medicine Unit, 84131 Salerno, Italy; tiziananotari7@gmail.com; 3 Department of Chemical Sciences, University of Naples Federico II, Via Cinthia, 21, 80126 Naples, Italy; marco.trifuoggi@unina.it (M.T.); antonella.giarra@unina.it (A.G.); 4 Department of Biological, Chemistry and Pharmaceutical Sciences and Technologies, University of Palermo, Viale delle Scienze Ed.16, 90128 Palermo, Italy; liana.bosco@unipa.it; 5 Andrology Unit of the “S. Francesco d’Assisi” Hospital, Local Health Authority (ASL) Salerno, EcoFoodFertility Project Coordination Unit, 84020 Oliveto Citra, Italy

**Keywords:** human spermatozoa, human protamines, Land of Fires, DNA oxidative damage, protein-DNA binding, heavy metals, EMSA, transgenerational effects

## Abstract

In our previous work, we reported alterations in protamines/histones ratio, in DNA binding of these proteins and their involvement in DNA oxidative damage in 84% of the young men living in the Land of Fires. In the present work, we extended our findings, evaluating any alterations in spermatozoa of a family case, a father and son, living in this area, to also give a first look at the possibility of transgenerational inherited effects of environmental contaminants on the molecular alterations of sperm nuclear basic proteins (SNBP), DNA and semen parameters. In the father and son, we found a diverse excess of copper and chromium in the semen, different alterations in SNBP content and low DNA binding affinity of these proteins. In addition, DNA damage, in the presence of CuCl_2_ and H_2_O_2_, increased by adding both the father and son SNBP. Interestingly, son SNBP, unlike his father, showed an unstable DNA binding and were able to produce DNA damage even without external addition of CuCl_2_, in line with a lower seminal antioxidant activity than the father. The peculiarity of some characteristics of son semen could be a basis for possible future studies on transgenerational effects of pollutants on fertility.

## 1. Introduction

A great concern has been raised for human health and environment in the well-known “Land of Fires”, in Campania (Southern Italy) [1,2]. In fact, in 2013–2014, this area, located on the northern-east side of the Campania region, was involved in illegal activities, including the uncontrolled spilling of industrial and urban waste and burning of toxic waste of various types, with release into the environmental of heavy metals and toxic chemicals that are detrimental for human health [3,4]. Over the past few decades, accumulated evidence has indicated that environmental pollution can also have a serious impact on the fitness, reproduction and survival of living organisms [5]. Pollution can increase infertility, and recent studies demonstrated that pollutants have diverse negative impacts on human spermatozoa. They, in fact, can alter not only the classic spermatozoa parameters, such as morphology, vitality, count and motility, but can also cause DNA fragmentation [6], and it would seem that reactive oxygen species (ROS) imbalance and related oxidative stress could be the principal cause through which pollutants can alter these parameters [6,7,8]. As a matter of fact, spermatozoa are very susceptible to environmental changes, mainly due to the pro-oxidant effects of environmental pollutants due not only to the limited volume of the cytoplasmic space, with less antioxidant [9] defense, but also because sperm membrane lipids are the target of reactive oxygen species (ROS). For these reasons, these cells are considered “ideal” bioindicators of environmental pollution and early sentinels of human health [10], and in our recent studies we provided new insights on DNA oxidative damage mechanisms in these cells. In particular, we discovered, through molecular investigations, that in young men living in the “Land of Fires”, some environmental pollutants can alter the properties of the sperm nuclear basic proteins (SNBP), making these proteins able to induce DNA breakage [11]. As is well known, in humans, during spermiogenesis, the majority of the somatic histones are replaced first by testis-specific histone variants and transition proteins and subsequently by protamines, to pack the genome into the highly condensed sperm nucleus [12,13]. In human spermatozoa, there are two types of protamines, named P1 and P2 [14,15], which occur normally in a strictly regulated 1:1 ratio [16]. In particular, a lot of studies demonstrated that strict regulation of protamines P1/P2 ratio, normally falling in the range 0.8–1.2, and a canonical protamines/histones ratio are critical for the fertility status of human sperm [17], considering that 10%–15% of histones in human are retained [18]. In our previous study on spermatozoa of young men living in the “Land of Fires”, we found that only 16% of the recruited subjects presented the canonical protamines/histones ratio, while the majority (about 62%) showed only histones and the remaining part (22%) had a non-canonical protamines/histones ratio [11]. It is well known that, as a result of industrialization, there has been a rapid increase in the variety of environmental pollutants which have negative repercussions on reproductive health. Moreover, several epidemiologic studies reported unexplained father–son effects from a variety of occupational or environmental exposures. Taking also into account that increasing evidence has shown that exposure to various pollutants can produce transgenerationally inherited effects and studies conducted on mice indicated that the susceptibility to certain pollutants can increase from one generation to the next [19], in the present work we deepened our studies, focusing on a family case living in the “Land of Fires”, constituted by a father (50 years old) and his son (18 years old). In these subjects, we analyzed the content of SNBP, the binding of these proteins to DNA and their ability to protect DNA from the action of free radicals. In addition, we also evaluated the potentiality of SNBP to promote the breakage of DNA, the antioxidant activities in the semen and the content of copper and chromium, two heavy metals, involved in Fenton reaction. Finally, we also took a first look at the possibility of possible transgenerational hereditary effects of environmental contaminants on molecular alterations of basic sperm proteins (SNBP), DNA and seminal parameters of these two subjects, with the aim of having a starting point for future, more extensive studies, with a larger sample of fathers and sons living in the “Land of Fires”.

## 2. Results

### 2.1. Characteristics of Impacted Areas Used for the Recruitment

The father and his son live in the “Land of Fires”, a high environmental impact area in Southern Italy (Campania region), while the control subjects (L-groups) belong to the low environmental impact area known as “Alto-medio Sele” (https://www.arpacampania.it/). For this study, we used two control subjects: control 1 (18 years old) and control 2 (50 years old). The geographical areas of residence of the analyzed subjects have already been described in Lettieri et al., 2020 [11]. These areas differ for the number of sites recognized by the Campania Region Environmental Protection Agency for the presence of a high concentration of toxic contaminants [20].

### 2.2. Aniline Blue Staining

**Figure 1:**
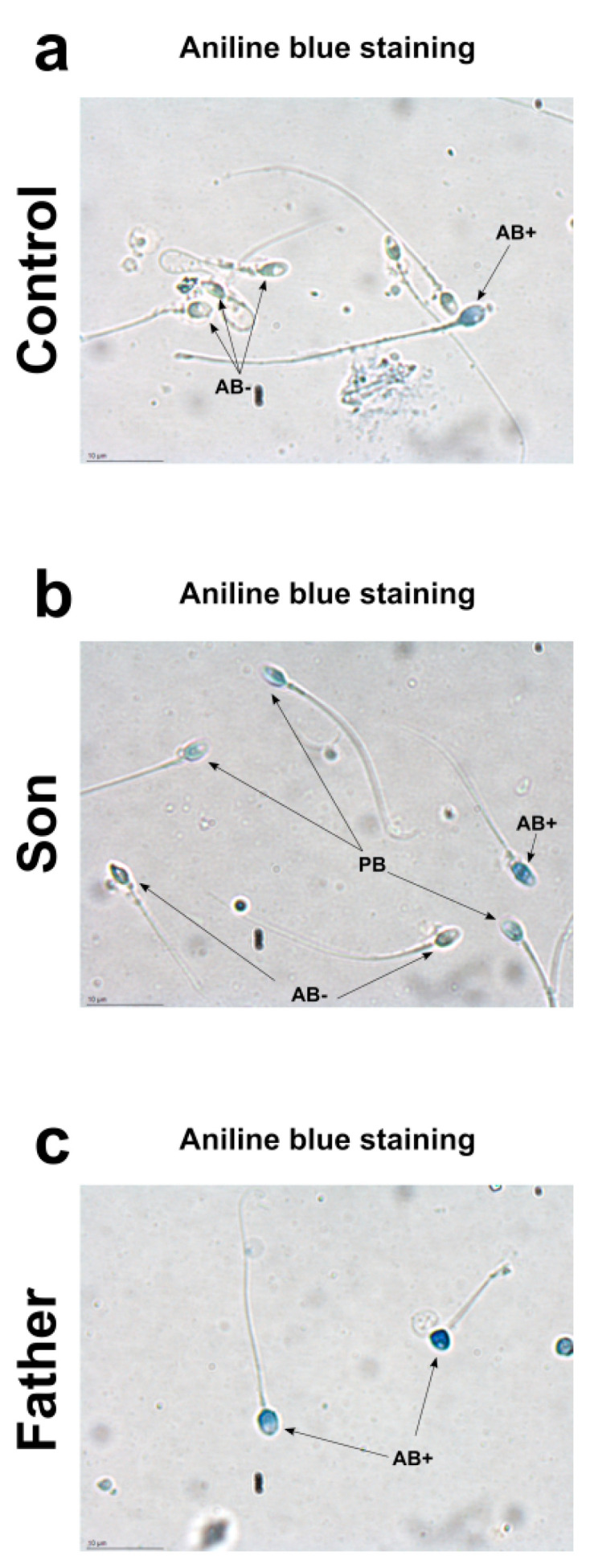
Aniline blue staining from control 1 sample (**a**), son (**b**) and father (**c**).

Aniline blue stain is a specific technique able to discriminate between lysine-rich histones and arginine/cysteine-rich protamine. Aniline selectively binds the lysines on the histones, giving to the cells the typical blue stain. A sperm with a complete nuclear maturation undergoes the almost total replacement of histones with protamine and it is negative to aniline blue staining (AB−). On the contrary, a sperm that has not completed the process of replacing histones with protamine is colored pale blue (PB) or dark blue (AB+), depending on the amount of histones still present. Regarding the nuclear maturity, AB−, PB and AB+ spermatozoa represent mature, moderately immature, and severely immature spermatozoa, respectively. Aniline staining of spermatozoa showed different results in the control, son and father samples (Figure 1). In the control 1 sample, the prevalence of aniline negative stained (AB−) sperms and the presence of only very few aniline blue stain positive (AB+) sperms was observed (a). Similar results were obtained in control 2 (Figure S1). The prevalence of pale blue (PB) aniline stained sperms were observed in the son sample (b). Differently, in the father sample, aniline blue stain-positive sperms (AB+) were observed (c).

### 2.3. Analysis of SNBP

**Figure 2:**
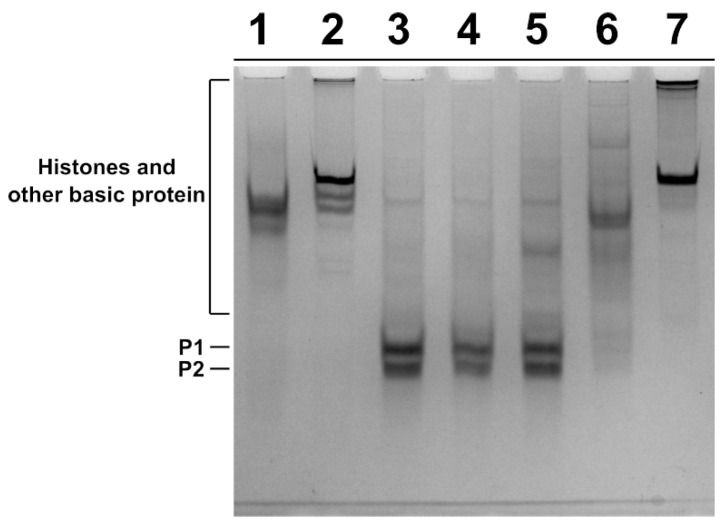
Characterization of human SNBP from samples belonging to two individuals of L-group (controls) and a father and his son living in the “Land of Fires”. AU-PAGE of SNBP of: rabbit total histones(lane 1); sea urchin total histones (lane 2); human control 1 and 2 samples, showing the CP/Hr (lanes 3–4); son sample showing the nCP/Hr (lane 5) and father sample showing only-H (lane 6); calf thymus H1 histone (lane 7). SNBP: sperm nuclear basic proteins; L-group: low impact area group; CP/Hr: canonical protamines/histones ratio; nCP/Hr: non-canonical protamines/histones ratio; only-H: only histones and other basic proteins.

SNBP were extracted from spermatozoa of the father and son and from two subjects living in the low impact area (L-group) and used as controls, and the protein content was characterized by acid-urea polyacrylamide gel electrophoresis (AU-PAGE) (Figure 2). In lanes 3 and 4, the electrophoretic pattern of control 1 and 2 samples, respectively, belonging to the L-group is shown. In these samples, we observed the classic electrophoretic pattern of human SNBP, with the canonical protamines/histones ratio (CP/Hr), which was accordingly previously described [21]. In the samples of the father and son, we observed several differences in the electrophoretic protein patterns. In fact, the SNBP of the father, shown in lane 6, consisted of only histones and other basic proteins (only-H) in which protamines seem to be absent; while the SNBP of the son showed the presence of both protamines and histones but not in the canonical ratio (nCP/Hr), (lane 5). In lanes 1, 2 and 7, instead, the electrophoretic patterns of rabbit total histones, sea urchin total histones and calf thymus H1 histone are shown, respectively.

### 2.4. DNA Binding Ability of SNBP Analyzed by Electrophoretic Mobility Shift Assay (EMSA)

**Figure 3:**
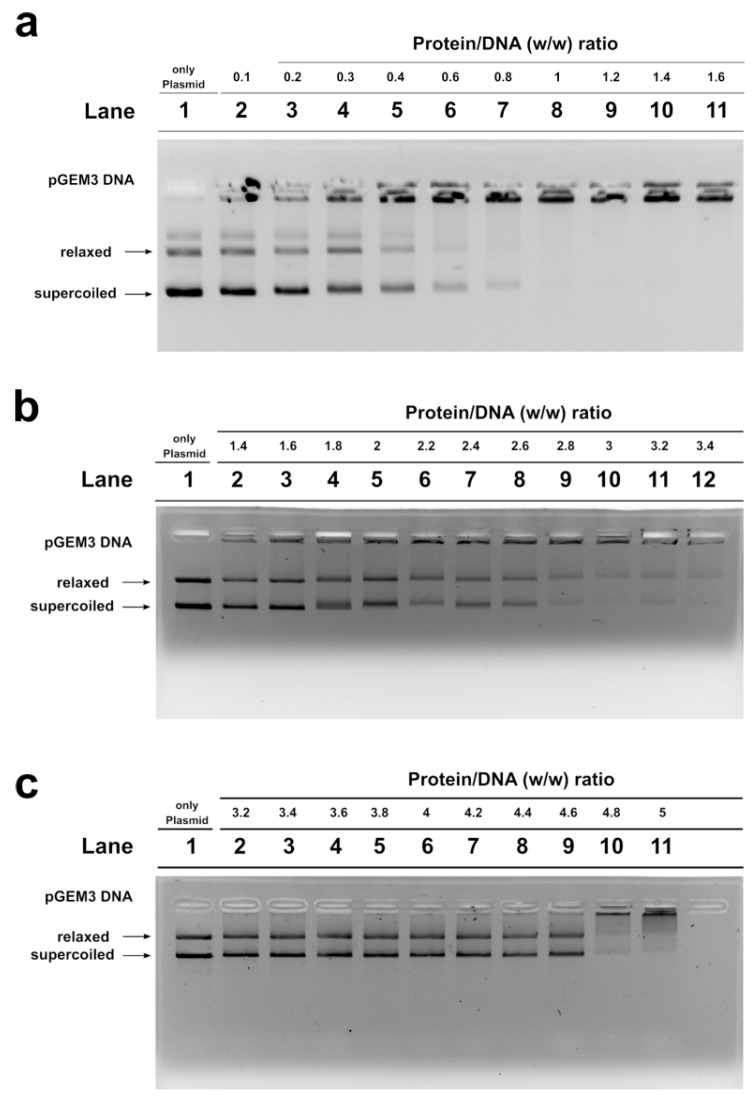
DNA binding ability of SNBP obtained from: control 1 (**a**); son (**b**) and father (**c**) analyzed by EMSA on 1% agarose gel. Bands on gel representing the state of pGEM3 plasmid DNA incubated in a w/w ratio with increasing amount of SNBP from samples containing: CP/Hr (**a**); nCP/Hr (**b**); only–H (**c**). Panel a from supplementary material of Lettieri et al., 2020 [11]. L-group: low impact area group; EMSA: electrophoresis mobility shift Assay; SNBP: sperm nuclear basic proteins; CP/Hr: canonical protamines/histones ratio; nCP/Hr: not canonical protamines/histones ratio; only-H: only histones and other basic proteins.

We studied, by electrophoretic mobility shift assays (EMSA), the differences in the ability to bind the DNA of the SNBP samples belonging to the control 1 subjects and of the father and son. In particular, we evaluated the proteins/DNA ratio required for obtain DNA saturation, detectable by the formation of a high-molecular weight DNA band, close to the well, in electrophoretic pattern [22]. All the analyzed SNBP samples interacted with DNA in the typical “all or nothing” DNA binding mode of SNBP in agreement with data previously reported for SNBP [23,24]. We found that SNBP samples presenting the CP/Hr profile belonging to the L-group (controls 1 and 2) reached DNA saturation at a protein/DNA ratio of about 1 (Figure 3a, lane 8 and Figure S2, lane 8, respectively).

SNBP samples from the son, instead, showed not only a reduced DNA binding ability, because DNA saturation did not occur even at the proteins/DNA ratio of about 3 (Figure 3b, lane 10), but also presented a not-stable binding mode to DNA. In fact, as shown in Figure 3b, we observed, at the protein/DNA ratio from 1.4 to 3 (lanes 2–10), a decrease in supercoiled and relaxed plasmid DNA fractions and an increase in the fraction close to the well. The next addition of proteins at protein/DNA ratio 3.2 (lane 11) resulted in a slight increase in supercoiled and relaxed DNA fractions, suggesting protein detachment to DNA (compare lanes 10 and 11 in Figure). In addition, a different binding mode to DNA appeared to be, due to the high-molecular weight DNA band, close to the well, in the electrophoretic pattern, which tended to decrease at higher protein/DNA ratios (lanes 11–12). As expected, SNBP samples from the father, presenting the only-H profile, showed low DNA binding ability; in fact, DNA saturation occurred at a 5 proteins/DNA ratio (Figure 3c, lane 11).

### 2.5. Trace Elements in Semen

**Figure 4:**
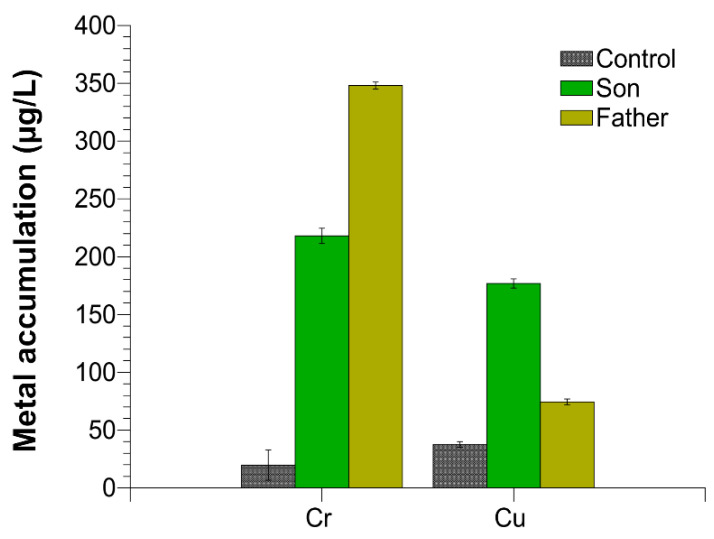
Chromium and copper accumulation in the semen of control 1, son and father.

We evaluated the accumulation of chromium and copper in the semen of the father, son and control 1. Control 2 presented similar values respect to control 1. We found an excess of these two metals in both the father and the son semen with respect to the controls, but the amount of the single metals was different between the father and son. In particular, as shown in Figure 4, chromium was about 17 and 10, while copper was about two and six times higher than the control in the father and son, respectively.

### 2.6. H_2_O_2_- Induced DNA Breakage in the Presence of Human SNBP

**Figure 5:**
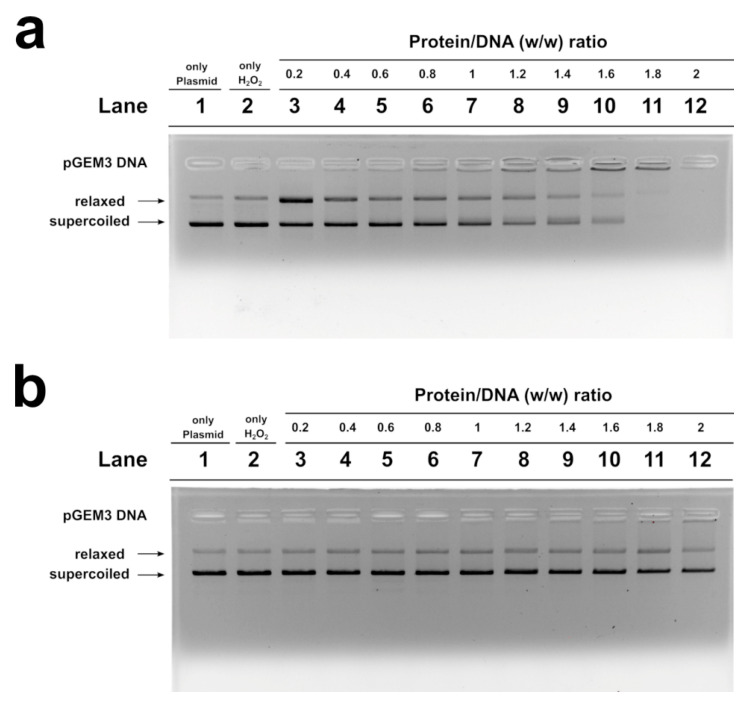
Analysis on 1% agarose gel of pGEM3 plamid DNA breakage induced by H_2_O_2_. In the presence of SNBP of the son (**a**) and father (**b**). SNBP: sperm nuclear basic proteins.

In Figure 5, the results of the analyses of H_2_O_2_-induced DNA breakage in the presence of control, son and father SNBP are shown. DNA breakage was evaluated by the conversion of supercoiled to relaxed form of pGEM3 DNA plasmid in presence of SNBP. In our experimental conditions, DNA breakage was not observed when the plasmid was mixed with 30 µM H_2_O_2_, a higher concentration of H_2_O_2_ µM, of at least 100, being necessary in order to observe the breakage of DNA (Figure S3).

The analysis performed with the samples of males living in low environmental impact areas (controls), and presenting protamines and histones in a canonical ratio, did not produce DNA breakage (Figures S4 and S5), while the addition of the son SNBP, containing nCP/Hr, at low protein/DNA ratios (0.2), to the pGEM3 DNA plasmid in the presence of H_2_O_2_ resulted in an increase in the relaxed plasmid DNA fraction at the detriment of the supercoiled one (Figure 5a, lane 3). Differently, the addition of the SNBP, extracted from father spermatozoa, presenting only-H, did not cause DNA breakage, providing a very similar result to that obtained by using control SNBP (Figure 5b).

### 2.7. DNA Protection Analysis

**Figure 6:**
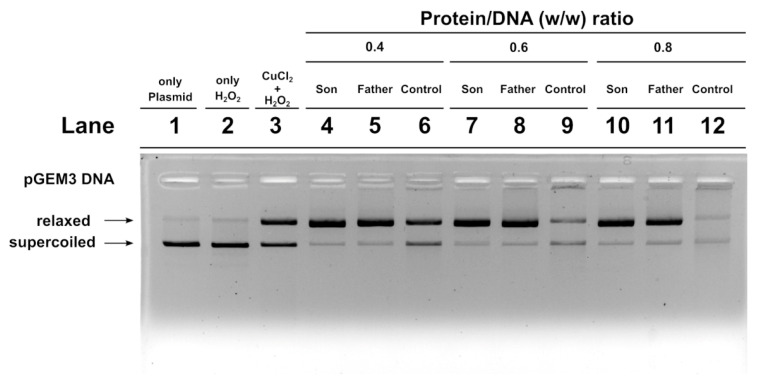
DNA protection analysis on 1% agarose gel of pGEM3 plasmid DNA in the presence of increasing (0.4, 0.6 and 0.8) controls (lanes 6, 9 and 12), son (lanes 4, 7 and 10) and father (5, 8 and 11) SNBP/DNA ratios. SNBP: sperm nuclear basic proteins.

We also carried out an assay to determine the potentiality of SNBP to protect DNA from oxidative damage. We created a condition in which damage to plasmid DNA occurred. In this condition, the plasmid DNA was placed in the presence of 30 µM H_2_O_2_ and 5 µM CuCl_2_, so as to cause the Fenton reaction and produce DNA breakage. This condition is shown in lane 3 of the agarose gel shown in Figure 6. In this condition, more than 50% of the plasmid DNA was in the relaxed form. The addition of son or father SNBP to this mixture, in protein/DNA ratios of 0.4, 0.6 and 0.8, produced an increase in DNA breakage with respect to the damage condition shown in lane 3. In fact, in those conditions, almost all the plasmid DNA resulted in the relaxed form. When SNBP, from subjects living in the low environment impact areas and presenting a CP/H ratio, were added, completely different results were obtained. In fact, already at protein/DNA ratio of 0.4. the entity of DNA breakage was lower with respect to the damage condition (compare lanes 6 and 3 in Figure 6). At 0.6 and 0.8 protein/DNA ratios, DNA damage was not observed, suggesting that these SNBP produced complexes capable of protecting DNA. In fact, in this latter case, at increasing SNBP/DNA ratios, the plasmid DNA bands corresponding to supercoiled and relaxed became less intense as DNA saturation took place, detectable by the appearance of a high molecular weight DNA band close to the well.

## 3. Discussion

Living organisms are continuously exposed to numerous pollutants that can influence numerous physiological functions, including reproduction [25]. In human sperm, the highly compacted sperm chromatin structure is, in fact, achieved thanks to the association of DNA mainly with protamines, highly basic proteins which are extremely rich in arginines residues and which comprise approximately 85–90% of the human sperm DNA [18]. For a correct structure of sperm chromatin is essential not only the right content of SNBP in spermatozoa but also that these proteins maintain their properties. In the “Land of Fires”, we recently found alterations in the protamines/histones ratio in the 84% of young man [11].

In the present work, we focused on a family case, constituted by a father and his son, living in the “Land of Fires”, and two controls, age-matched father and son, from low environmental areas. We started from the observation of different percentages of aniline blue positive sperm stained cells in these subjects. The controls, son and father were representative of the three categories of subjects reported in Lettieri et al., 2020 [11]. Therefore, we analyzed the possible alterations of SNBP and DNA of these subjects at molecular level. As expected, by AU-PAGE analyses, the spermatozoa of controls showed a CP/H ratio—those of father contained only histones, while protamines and histones, but not in the canonical ratio, were found in the son. We also evaluated the DNA interaction of these SNBP, by EMSA, determining the proteins/DNA ratio necessary for DNA saturation, indicated by the formation of a high molecular weight DNA band, close to the well, in electrophoretic pattern. Generally, in spermatozoa of men presenting a canonical protammines/histones ratio and living in low environmental areas, we found, in our previous studies, that DNA saturation was achieved at a SNBP/DNA ratio of about 1 [11]. In EMSA experiments, we encountered a low DNA binding affinity for SNBP of both the father and son. For SNBP of the latter, an unstable bond with DNA was also observed and we noted that at the higher protein/DNA ratios tested, the high molecular weight DNA band, close to the well, was never clearly visible, suggesting the formation of different DNA–SNBP aggregates. The data obtained on the father and son indicated that the son belongs to the 22% of young men presenting a nCP/H ratio, as tested in Lettieri et al., 2020 [11]. The father, instead, presented only-H in spermatozoa, as with the majority of the samples analyzed in Lettieri et al., 2020 [11]. Father SNBP, as expected, showed a very low DNA binding affinity because of the lower basicity of histones with respect to protamines. The high level of compaction of the genome, due to protamines, confers protection from the effects of genotoxic factors, and optimizes the aerodynamic spermatozoan shape, useful for correct motility [26,27]. The electrophoretic analysis of the SNBP of the controls, father and son samples, resulted in a line with aniline blue staining.

Male gametes, for their continuous production and for exposition to environmental agents, such as oxidizing agents, are the most sensitive cells to the accumulation of damaged DNA [28,29,30,31]. Our research group has widely documented that some heavy metals are able to produce alterations in some cells [32] and to change the properties of some proteins [33], among which include human and marine organisms’ SNBP [11,34,35]. In particular, in these latter proteins, some heavy metals can reverse their canonical protective rule, making them able to participate in DNA breakage [11,34,36,37]. In addition, our studies also indicated that in organisms exposed to environments polluted by heavy metals, we found accumulation of some of these metals in gonadal tissues, semen and SNBP [37,38,39]. As a matter of fact, in the semen of the father and son, we found different excesses of copper and chromium, two heavy metals involved in the Fenton reaction [40]. As shown in Figure 4, chromium was about 17 and 10, while copper was about two and six times higher than the control in the father and son, respectively. So, after determining the accumulation of chromium and copper in the semen of these subjects, we analyzed the potentiality of their SNBP to protect DNA or induce DNA oxidative damage.

We did not observe DNA breakage in the presence of H_2_O_2_ when added to plasmid DNA of both the controls and father SNBP. In contrast, son SNBP produced DNA breakage at 0.2 protein/DNA ratio. We also analyzed the ability of the SNBP of the father and son to protect DNA. We prepared a mixture containing plasmid DNA together with appropriate amount of copper chloride and hydrogen peroxide in order to induce a Fenton reaction. To this mixture we added the SNBP of controls, the father or son and obtained different results. In particular, while control SNBP were able to aggregate DNA and prevent DNA oxidative damage, the adding of son or father SNBP produced a similar increase in the fraction of the relaxed form of plasmid DNA, indicative of major DNA damage with respect to the control mixture. The ability of son SNBP to produce oxidative DNA damage without external adding of copper could be explained considering that in the semen of the son, an excess of copper and chromium was found, but especially of copper. In addition, son SNPB are constituted mainly by protamines and several studies demonstrated that copper can form several binary and ternary complexes with arginine residues [41,42,43], of which human protamines are extremely rich, promoting a site-specific damage at guanine residues of DNA by a selective binding between guanine and arginine [44]. Moreover, the mediation of oxidative DNA damage by copper (II) complexes with the N-terminal sequence of human protamine P2 has been reported [45]. The involvement of SNBP of both son and father in DNA oxidative damage in the presence of H_2_O_2_ and copper and the more marked extent of DNA oxidative damage in the assays in which we added the SNBP of these subjects to a mixture containing plasmid DNA, copper and hydrogen peroxide could be ascribed to the concomitant presence in the semen of these subjects of an excess of chromium and copper, able to bind histones and protamines, respectively. The binding between histones and chromium happens through lysine residues [46,47] and could cause DNA damage in a similar manner as hypothesized for copper, since the toxic effect of this metal results in radical-mediated DNA strand breakage and the formation of stable chromium–DNA complexes, together with chromium–DNA adducts and protein–chromium–DNA and DNA–chromium–DNA cross-links [48,49]. The unstable DNA bond observed only for son SNBP could be due to the excess of copper, which can also determine conformational change in these proteins, as already demonstrated for protamine-like of mussels exposed to this metal [37,39]. Moreover, the presence of nCP/Hr ratio could also produce a competition between these two types of proteins for DNA binding. Taking into account that in human sperm chromatin, 10–15% of histones are retained [18,50,51,52], forming a heterogeneous mixture of nucleohistones and nucleoprotamines, we could hypothesize that the presence of both protamines and histones in altered ratios, in the case of the son, could determine not only an unstable binding to DNA, but also a reduced DNA protection to the external agents, such as heavy metals. There is also a need to consider that growing evidence suggests that epigenetic effects may in part be responsible for the genotoxicity and carcinogenicity of chromium [53,54,55,56], also taking into account that long-term chromium exposure may cause a significant increase in histone deacetylation. This effect certainly could affect the histones–protamines transition, which requires histone acetylase activity, and thus could explain the presence of only histones in the father spermatozoa. After all, chromium, the other heavy metal found in excess in the semen of these subjects, but in particular in the father, is also able to participate in Fenton-like reactions producing reactive oxygen species. This metal could influence the structure of chromatin by binding to both DNA and histones [46,56]. In this study, the seminal parameters and the redox status were also evaluated. Table 1 shows the data regarding the seminal parameters of the controls, father and son samples. These analyses show a poor quality of father and son semen with respect to the controls. 

Interestingly, a lower antioxidant activity in the son, even more than the father, was observed, indicative of a reduced ability to counteract oxidative stress (Table 2).

Given the dramatic increase in both the number and complexity of environmental chemical contaminants during the last several decades, our results, together with those previously published, highlight how environmental pollution can produce a deterioration in semen quality. 

In conclusion, in the present work, having considered only a family case, the peculiarity of some characteristics of son semen could represent a starting point for possible future studies on transgenerational effects of pollutants on fertility, using wider sampling. After all, a study conducted on the semen quality of young adult intracytoplasmic sperm injection ICSI offspring demonstrated that the worldwide oldest ICSI-conceived adults presented significantly lower sperm concentration, lower total sperm count as well as lower total motile sperm count in comparison to a control group of spontaneously conceived peers [57]. In clinical practice when there are no conditions that indicate low sperm quality according to the classical parameters (number, motility, morphology and fragmentation state of sperm DNA), i.e., when we refer to idiopathic infertility, although there are several functional tests on the sperm, defining with certainty the fertile capacity of semen is not possible, considering further the interactions with the female part. Therefore, based on our study, the possible implications for fertility could relate to the risk of reduced reproductive capacity in the future. In fact, considering that, it is not always possible to define the state of fertility of an individual on the basis of the classic parameters of the spermiogram which could now be normal, but not subsequently; as a result, these individuals could be in a borderline condition, also taking into account that in the last 45 years, there has been a decline in semen quality. In fact, a systematic review and meta-regression analysis [58] demonstrated that sperm concentration (SC) and total sperm count (TSC) has declined significantly among men from North America, Europe and Australia between 1973 and 2011. In particular, the mean SC and TSC declined, on average, by1.4% and 1.6% per year, with an overall decline of 52.4% and 59.3%, respectively. In addition, a retrospective study reported a significant and strong decline in sperm concentration and morphology in the whole of France between 1989 and 2005 [59]. In fact, ubiquitary exposure to chemicals has been growing in the general population of France since the 1950s [59]. In particular, main endocrine disruptor chemicals (EDCs) have been found in biological matrices of French people [60], some (non-dioxin-like PCB, pesticides and triclosan) at a level higher than that in other countries. These results could therefore be consistent with the endocrine disruptor hypothesis, recently strengthened in an international report [61]. A similar situation has also been reported in the “Land of Fires”, where, during the last few decades, the increasing occurrence of disorders of the male reproductive system in humans has raised attention about the possible environmental risk factors [10]. Therefore, the global decrease in sperm concentration and morphology seems most probably to be due to global changes in environmental exposure in specific areas. Therefore, it is very important to also consider the transgenerational effects that could have influenced this rapid decline in semen quality. In particular, the alterations observed in the son with our molecular approaches combined with the alterations of the redox state of his semen, as well as the excess of metals, could lead to a greater consumption of antioxidants and therefore favor a possible condition of oxidative stress, responsible for the observed alterations through these molecular approaches. Taking into account the transgenerational hypersensitivity mechanisms found in mice [19], it would also be possible to hypothesize that in the son, the antioxidant and detoxifying enzyme systems may be less efficient due to the genetic and/or epigenetic pathways induced by the paternal gametes [62]. Therefore, as future perspectives of this study, we intend to do a more extensive one, with a larger sampling of fathers and sons resident in the “Land of Fires”, to support our observations, which, however, want to represent a call to attention for a greater possible reproductive risk that requires further studies. The implications on the future reproductive capacity of young people living in this area, which for decades has shown very high pollution rates, which are reflected on the health of the resident population [63], cannot therefore be underestimated, especially if we consider that semen seems to represent an excellent marker of environmental health and general health [64,65,66,67]. 

## 4. Materials and Methods 

### 4.1. Reagents

All used reagents were of analytical grade and purchased at Sigma-Aldrich (Merck KGaA, Darmstadt, Germany).

### 4.2. Ethical Statements

All methods were carried out in accordance with the Code of Ethics of the World Medical Association (Declaration of Helsinki) guidelines and regulations. All experimental protocols were approved by the Ethical Committee of the Local Health Authority Campania Sud-Salerno (Committee code n. 43 of 30/06/2015). Informed consent was obtained from the recruited subjects (over 18) before sample collection.

### 4.3. Recruitment

The semen samples of the father (50 years old) and son (18 years old) came from Casalnuovo, a municipality belonging to the “Land of Fires” and were obtained by Medicina Futura center (Acerra-Province of Naples). The “Land of Fires” is a high environmental impact area in Southern Italy (Campania region). According to the Campania Region Environmental Protection Agency report (https://www.arpacampania.it/), Campania, the “Land of Fires”, is officially recognized as an area with the highest concentration of environmental disposal sites of toxic waste often associated with their combustion. Semen samples from controls came from San Francesco d’Assisi Hospital in Oliveto Citra-Province of Salerno, which is a municipality belonging to the low environmental impact area known as “Alto-medio Sele” (https://www.arpacampania.it/). The control sample used for this study belonged to the previous recruitment [11] and was representative of all semen samples presenting CP/Hr. For this study, the two control subjects considered were recruited as an age-matched father and son. In particular, control 1 was 18 years old, while control 2 was 50 years old. The economy of this area is mainly based on low-to-medium-scale farming and without known illegal disposal of toxic wastes. The samples analysis is part of a pilot study (EcoFoodFertility initiative, www.ecofoodfertility.it) which consists of the analysis of the effects produced by environmental pollution on humans, through the study of human semen that becomes an early biomarker of pollution in healthy men [6,10,68]. The father and son and control participated as volunteers at the study and they were selected according to the following criteria: residence for at least 10 years in the study area, no known chronic diseases (diabetes or other systemic diseases), no varicocele, no prostatitis, and other factors that could negatively affect semen quality (such as fever, medications, exposure to X-rays, etc.), non-drinkers, non-smokers, no reported history of drug abuse, and no known occupational exposure to toxic chemicals). In addition, the participants follow a healthy diet and practice at least 30 min of walking at day. Data were collected by questionnaire and physical examination, including the urogenital evaluation (testis volume and transrectal prostate evaluation was made). The recruiting andrologist (the recruiter) assigned a code number to each volunteer in order to preserve anonymity. Each code number was uploaded into a computer database together with personal and clinical information.

### 4.4. Semen Quality Evaluation

Parameters of semen quality were assessed by the examining andrologist (the evaluator), according to the World Health Organization (WHO) guidelines [69]: semen volume, pH and sperm concentration, motility and morphology were evaluated.

### 4.5. RedOx Status Evaluation in Seminal Plasma

TAC was measured by using the Antioxidant Assay Kit (Cayman Chemical, Ann Arbor, MI, USA), following the manufacturer’s instructions. The determination of total and oxidized glutathione (GSH + GSSG and GSSG, respectively) was spectrophotometrically measured (at 412 nm) accordingly to the Ellman’s method [70] and their concentration—upon normalization to protein content—was expressed as nmoles/mg protein.

### 4.6. Spermatozoa Collection and Sperm Proteins Extraction

In order to separate the spermatozoa from seminal plasma, the semen samples were centrifuged for 30 min at 5500× *g* at 4 °C and sperm pellets were stored at −80 °C in aliquots of 50 µL. Protamines extraction from spermatozoa was performed as described in Lettieri et al., 2020 [11]. In brief, the sperm pellets were washed twice with 500 μL of phenylmethanesulfonyl fluoride (PMSF), centrifuged at 10,480× *g* for 5 min at 4 °C, resuspended with 50 μL of 1 mM PMSF and 50 μL of a solution containing 6 M guanidinium chloride and 10 mM DTT and then incubated at 20 °C for 30 min. Afterwards, the samples were added with 5 volumes of cold ethanol 100% and incubated at −20 °C for 60 min to obtain the sperm chromatin precipitation. The samples were centrifuged at 13,680× *g* for 15 min at 4 °C and the pellet obtained was resuspended in 500 μL of 0.5 M HCl to solubilize SNBP. The samples were incubated for 5 min at 37 °C and then centrifuged a 1000× *g* for 10 min at 4 °C. At the end of this step, at the supernatant, trichloro acetic acid (TCA), was added for a final concentration of 20%, in order to precipitate the SNBP. The samples were incubated 60 min at 4 °C, and then centrifuged at 14,000× *g* for 10 min at 4 °C. The pellet obtained, containing SNBP was washed twice with 500 μL of acetone containing 1% β-mercaptoethanol, centrifuged twice at 14,000× *g* for 10 min at 4 °C and the final pellet was dried in a speed-vacuum for 10–15 min. The dried proteins were resuspended in 50 μL of ultrapure water (milliQ) and stored at −20 °C in aliquots of 50 µg.

### 4.7. Acid-Urea Polyacrylamide Gel Electrophoresis of SNBP

Acid-urea polyacrylamide gel electrophoresis (AU-PAGE) was used to analyze human SNBP as previously described in Soler-Ventura et al., 2018 [21] but with a few modifications reported in Lettieri et al., 2020 [11]. The composition of the gel for a volume of 8 mL was: 15% acrylamide/0.1% N,N’-Methylene-bis-acrylamide, (optimal acrylamide/Bis-acrylamide ratio for the separation of human SNBP), 2.5 M urea, 0.9 M acetic acid, 80 μL of TEMED and 140 μL of 10% APS. 

After the electrophoresis, gels were stained with Amido Black, and then with Coumassie Blue Brilliant R-250 as previously described [71]. Gels were acquired using a Gel-Doc system (BioRad Hercules, CA, USA) by Quantity One v.4.4.0 (BioRad, Hercules, CA, USA) software. The software ImageJ ver 1.50 d (https://imagej.nih.gov/ij/) supported by the National Institute of Health (Bethesda, Maryland, USA) was used for densitometric analyses of the bands on the gel.

### 4.8. Plasmid DNA Preparation

pGEM3 plasmid (2867 bp) was extracted from transformed *Escherichia coli* HB 101 cells, using the standard protocol of the QIAGEN Plasmid Midi Purification kit (QIAGEN Plasmid Midi Purification handbook, third edition© 2020, Hilden, Germany), but following the precautions described in Carbone et al., 2012 [72], in order to obtain high amounts of supercoiled pDNA. In order to evaluate the quality of plasmid DNA, gel electrophoresis on 1% agarose gels in 89 mM Tris-HCl pH 8.0, 2 mM EDTA and 89 mM boric acid (TBE) was used. The circular form of the plasmid pGEM3 was used for electrophoretic mobility shift assays (EMSA) of DNA and for the analysis of DNA breakage and DNA breakage/protection by SNBP.

### 4.9. DNA Binding Affinity of SNBP by EMSA

The effect of human SNBP extracted from the father, son and control samples on DNA was analyzed by EMSA as previously described [73], with slight modifications. Mixtures of DNA/proteins were prepared. In all samples, a fixed amount of plasmid DNA (pGEM3) (150 ng) and increasing amount of SNBP were used in order to obtain protein/DNA *wt*/*wt* ratios between 0.1 and 5, as indicated in the section results. We evaluated the protein/DNA ratio necessary to achieve DNA saturation. In the preparation of the samples, the various components were added in the following order: ultrapure water (milliQ), DNA, proteins. After that, the samples were left for 5 min at room temperature to make the proteins and DNA interact, and then all samples were added with TBE 10 X (to obtain TBE 1X final concentration) just before running the gels and analyzed on 1% agarose gel in TBE. The electrophoretic run was performed on 1% agarose gel in TBE at 100 V for about 30 min at room temperature. At the end, DNA migration was visualized by staining agarose gels with ethidium bromide (2 µg/mL) after electrophoresis. All experiments were performed at least five times. Gels were acquired using a Gel-Doc system (BioRad, Hercules, CA, USA) through Quantity One v.4.4.0 (BioRad, Hercules, CA, USA) software. A densitometric analysis of the bands on the gel was performed using the software ImageJ ver 1.50 d (Wayne Rasband, National Institute of Health, Bethesda, ML, USA, https://imagej.nih.gov/ij/, 1997–2018).

### 4.10. Aniline Blue Staining

The aniline staining was performed as described by Pourmsumi et al. [74], with a few modifications. In brief, the fresh semen smear of each sample was air dried and then stained with 5% aqueous aniline blue stain (Histon Color Test, AB Analitica, Padua, Italy) in 4% acetic acid (pH 3.5) for 5 min. A cover slide 24 mm × 50 mm was put on each slide. Stained and unstained spermatozoa were observed using light microscopy (Nikon Eclipse Ci) at ×1000 magnification under oil immersion (Plan 100×/1.25 oil objective). A total of 200 cells were manually evaluated on each slide for the type of staining pattern: aniline blue positive (AB+) cells, pale blue (PB) cells and aniline blue negative (AB−) unstained cells.

### 4.11. DNA Breakage Analyses

pGEM3 plasmid DNA breakage in the presence of SNBP extracted from the father, son and control and 30μM H_2_O_2_ was analyzed on 1% agarose gel in TBE 1X final concentration. The preparation of the samples was performed as described by Piscopo 2019 [39]. In brief, into any samples we put ultrapure water (milliQ), plasmid DNA pGEM3 (a fixed amount of 150 ng) and SNBP extracted from the father, son or control in increasing *w*/*w* protein/DNA ratios in a range from 0.2 to 2. The samples were incubated at room temperature for 5 min to make proteins and DNA interact, after which H_2_O_2_ was added and the samples were incubated in a Thermoblock set at 37 °C for about 30 min in the dark. At the end of incubation, sample buffer 10X (1X final concentration in the samples) was added just before electrophoresis analysis, in order to avoid the EDTA coordination of eventual metals. The electrophoretic analysis of the samples was conducted on 1% agarose gel at 100 V for 30 min in TEB 1X. After electrophoresis, agarose gels were stained with ethidium bromide (2 μg/mL) in order to visualize DNA migration and then the images of the gels were acquired at the GelDoc Biorad (Hercules, CA, USA). All experiments were performed at least five times.

### 4.12. DNA Protection Analysis

SNBP ability to protect DNA from oxidative damage in the presence of 30 µM H_2_O_2_ and 5 µM CuCl_2_ was performed by using plasmid DNA (pGEM3) and SNBP extracted from the father, son and control samples. The samples were prepared using EMSA protocol described in the paragraph “DNA binding affinity of sperm proteins by EMSA”, with slight modifications. In particular, 150 ng of plasmid DNA (pGEM3) and proteins/DNA *w/w* ratios in a range from 0.4 to 0.8 were used. After 5 min of interaction, at room temperature, between DNA and SNBP, H_2_O_2_ and CuCl_2_ were added and the samples were incubated in the dark for 30 min in a Thermoblock set at 37 °C. At the end of incubation, sample buffer 10X (1X final concentration in the samples) was added just before electrophoresis analysis, in order to avoid the EDTA coordination of eventual metals. The electrophoretic analysis of the samples was conducted on 1% agarose gel at 100 V for 30 min in TEB 1X. After electrophoresis, agarose gels were stained with ethidium bromide (2 μg/mL) in order to visualize DNA migration and then the images of the gels were acquired at the GelDoc Biorad (Hercules, CA, USA). All experiments were performed at least five times.

### 4.13. Trace Elements in Semen

One milliliter of nitric acid (HNO_3_ ≥ 69%, *v/v* TraceSELECT^®^, fisher scientific, Waltham, Massachusetts, USA) was added to each tube containing aliquots of 600µL of semen and the suspension was subjected to oxidative acid digestion in a microwave system equipped with autosampler (CEM DISCOVER SP-D, CEM Srl, Cologno al Serio, Bergamo, Italy). 

The elemental analysis was conducted by inductively coupled plasma mass spectrometry (ICP-MS). Element concentrations were determined from a calibration curve calculated on the basis of five concentrations for each of analyzed elements obtained from certified standard solutions and the final concentrations were expressed in µg/L. The limits of detection and quantification (LOD and LOQ) were calculated considering, respectively, 3 and 10 times the standard deviation (SD) of 10 replicates made on a negative control. ICP-MS analyses were performed for the quantification of the following elements of copper and chromium.

## Figures and Tables

**Table 1:** Semen parameters in controls, father and son.

	Control 1		Control 2		Father		Son	
	Mean	SD	Mean	SD	Mean	SD	Mean	SD
Volume (mL)	2.9	±0.2	2	±0.4	2.7	±0.6	2.3	±0.6
Sperm Concetration (million/mL)	100.7	±1.5	83.0	±10.1	13.0	±7.8	67.7	±39.1
Total sperm	288.7	±23.1	168.1	±31.7	21.7	±10.8	138.1	±52.7
Total motility %	82.7	±2.5	79.3	±4.0	66.7	±15.3	63.3	±10.4
Progressive motility %	72.7	±2.5	66.0	±1.7	40.0	±18.0	48.3	±14.4
Normal form %	11.3	±1.2	13.3	±2.1	3.7 *	±0.6	13.0	±4.6

Data are presented as mean ± SD from triplicate analyses on semen samples. Asterisk (*) indicates the significance (*p* < 0.05) between the subject (father or son) and respective age-matched control. Control 1: 18 years old; control 2: 50 years old.

**Table 2:** Semen redox status.

	Control 1		Control 2		Father		Son	
	Mean	SD	Mean	SD	Mean	SD	Mean	SD
**TAC ^Δ^**	1.4	±0.1	1.3	±0.1	0.9 *	±0.1	0.7 *	±0.1
**GSH**	0.6	±0.1	0.5	±0.1	0.3	±0.1	0.2 *	±0.0
**GSSG**	0.2	±0.1	0.2	±0.0	0.1 *	±0.0	0.1	±0.0

Data are presented as mean ± SD from triplicate analyses on individual semen sample. Asterisk (*) indicates the significance (*p* < 0.05) between the subject (father or son) and respective age-matched control. Control 1: 18 years old; control 2: 50 years old; triangle (^Δ^) indicate the significance (*p* < 0.05) between father and son. TAC: total antioxidant capacity; GSH: reduced glutathione; GSSG: oxidized glutathione.

## Supplementary Materials

Supplementary Materials can be found at https://www.mdpi.com/1422-0067/21/18/6710/s1.

## Abbreviations

ROS	reactive oxygen species
TCA	trichloro acetic acid
AU-PAGE	acid-urea polyacrylamide gel electrophoresis
EMSA	electrophoretic mobility shift assays
EDTA	ethylenediaminetetraacetic acid
TEB	Tris-Borate-EDTA
SNBP	sperm nuclear basic protein
pDNA	plasmid DNA
DTT	dithiothreitol
L-group	man living in the low environmental impact areas
CP/Hr	canonical protamines/histones ratio
nCP/Hr	not canonical protamines/histones ratio
only-H	only histones and other basic proteins
TAC	total antioxidant capacity
GSH	glutathione
GSSG	oxidized glutathione
SC	sperm concentration
TSC	total sperm count
ICSI	intracytoplasmic sperm injection
