# Supplementary material for: Molecular Alterations in Spermatozoa of a Family Case Living in the Land of Fires—A First Look at Possible Transgenerational Effects of Pollutants
Source: Int J Mol Sci. 2020 Sep 13;21(18):6710. doi: 10.3390/ijms21186710 (PMC7555199; doi:10.3390/ijms21186710)
Supplement: Supplementary file 1 [file ijms-21-06710-s001.pdf]

Molecular Alterations in Spermatozoa of a Family Case Living in the Land of Fires. A First Look at Possible Transgenerational Effects of Pollutants

Gennaro Lettieri [1,†], Federica Marra [1,†], Claudia Moriello [1,†], Marina Prisco [1], Tiziana Notari [2], Marco Trifuoggi [3], Antonella Giarra [3], Liana Bosco [4], Luigi Montano [5,*] and Marina Piscopo [1,*]

[1] Department of Biology, University of Naples Federico II, 80126 Napoli, Italy; gennarole@outlook.com (G.L.); federicamarra14@gmail.com (F.M.); cla_mar97@hotmail.it (C.M.); marina.prisco@unina.it (M.P.)
[2] Check Up—Day Surgery, Polydiagnostic and Research Centre, Reproductive Medicine Unit, 84131 Salerno, Italy; tiziananotari7@gmail.com
[3] Department of Chemical Sciences, University of Naples Federico II, Via Cinthia, 21, 80126 Naples, Italy; marco.trifuoggi@unina.it (M.T.); antonella.giarra@unina.it (A.G.)
[4] Department of Biological, Chemistry and Pharmaceutical Sciences and Technologies, University of Palermo, Viale delle Scienze Ed.16, 90128 Palermo, Italy; liana.bosco@unipa.it
[5] Andrology Unit of the "S. Francesco d'Assisi" Hospital, Local Health Authority (ASL) Salerno, EcoFoodFertility Project Coordination Unit, 84020 Oliveto Citra, Italy
* Correspondence: l.montano@aslsalerno.it (L.M.); marina.piscopo@unina.it (M.P.); Tel.: +39-081-679-081 (M.P.); +39-082-879-7111 (ext. 271) (L.M.)
† These authors contributed equally to this work.

Supplementary Figures

Figure S1. Aniline blue staining.

Figure S2. DNA binding affinity of the control 2

Figure S3. Evaluation of pGEM3 DNA plasmid breakage in presence of H_2O_2

Figure S4. Analysis on 1% agarose gel of pGEM3 plamid DNA breakage induced by H_2O_2, in the presence of SNBP from control 1.

Figure S5. Analysis on 1% agarose gel of pGEM3 plamid DNA breakage induced by H_2O_2, in the presence of SNBP from control 2.

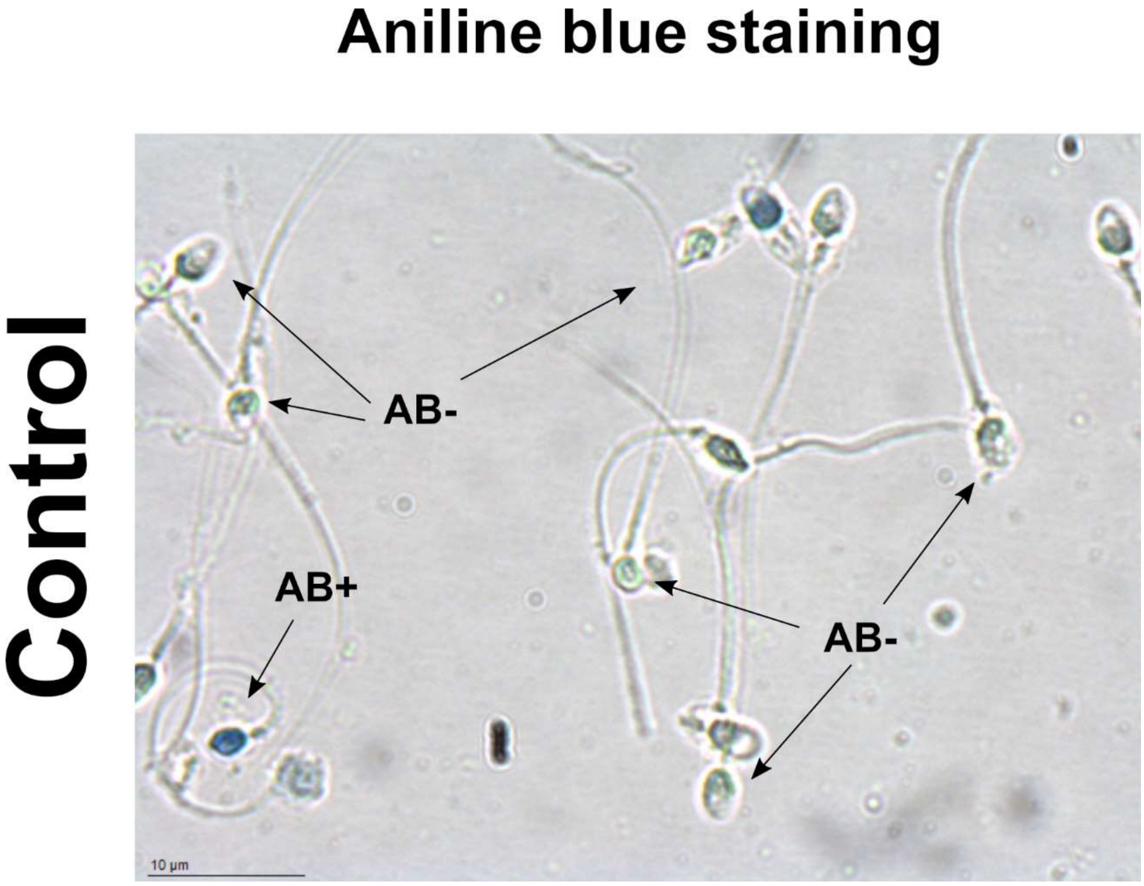

Figure S1. Aniline staining of control 2.

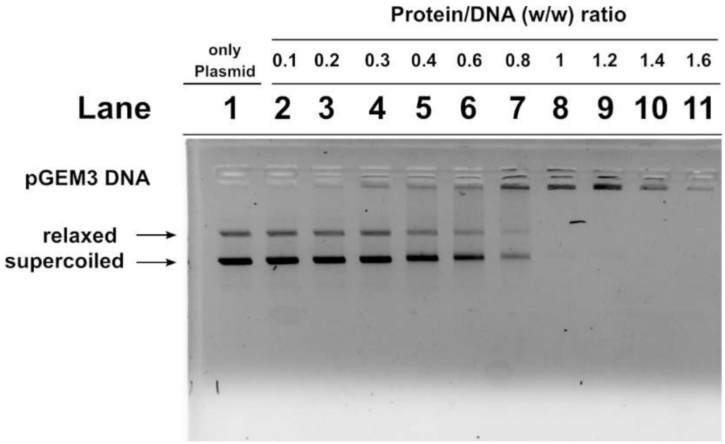

Figure S2. DNA binding ability of sperm proteins of control 2.

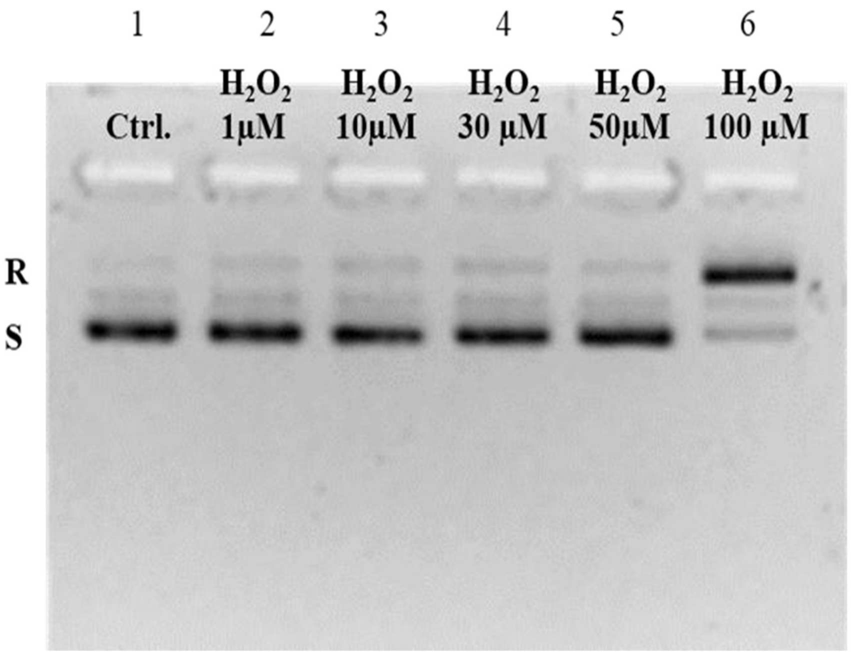

Figure S3. Evaluation of pGEM3 DNA plasmid breakage in presence of H_2O_2 concentrations. DNA breakage is evaluated by the conversion of supercoiled (S) to relaxed (R) form of 150 ng of circular pGEM3 DNA plasmid in absence (Ctrl.) and in presence of increasing concentration of H_2O_2 (from 1 to 100 µM)

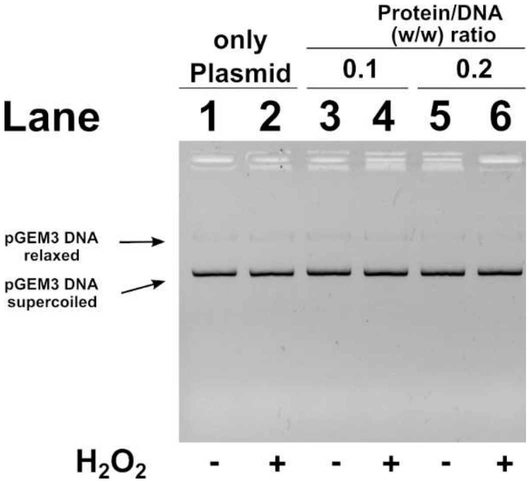

Figure S4. Analysis on 1% agarose gel of pGEM3 plamid DNA breakage induced by H_2O_2, in the presence of SNBP from control 1 samples showing the CP/Hr.

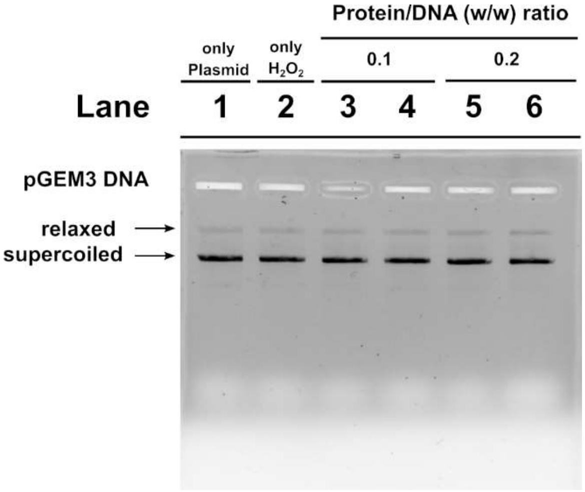

Figure S5. Analysis on 1% agarose gel of pGEM3 plamid DNA breakage induced by H_2O_2, in the presence of SNBP from control 2 samples showing the CP/Hr.
